# Rotavirus infections in the pediatric population: a comparative study of pre-COVID and COVID-19 pandemic periods

**DOI:** 10.3389/fpubh.2025.1495848

**Published:** 2025-01-29

**Authors:** Bahar Öztelcan Gündüz, Kazım Kutlutürk, Bülent Ünay

**Affiliations:** ^1^Gülhane Research and Training Hospital, General Pediatrics, Ankara, Türkiye; ^2^Gülhane Research and Training Hospital, Pediatric Neurology, Ankara, Türkiye

**Keywords:** rotavirus, COVID-19, gastroenteritis, pediatric infections, emergency department, hospitalization factors

## Abstract

**Background:**

This study investigates the impact of the COVID-19 pandemic on the incidence and clinical outcomes of rotavirus infections in the pediatric population.

**Methods:**

This retrospective study was conducted at the Pediatrics Clinic of Gülhane Research and Training Hospital, analyzing data from the pre-COVID-19 period (January 1, 2017 – January 1, 2020) and during the COVID-19 pandemic period (March 11, 2020 – August 31, 2022). Patient data, including demographic characteristics, presenting complaints, laboratory results, and hospitalization status, were collected from hospital records. Statistical comparisons were made to evaluate changes in rotavirus infection patterns between the two examined periods.

**Results:**

The data of a total of 3,915 pediatric patients with diarrhea were included, with 468 (6.8%) testing positive for rotavirus. Three hundred and forty of these cases (72.6%) were detected during the COVID-19 pandemic period, while 128 cases (27.4%) were detected before the pandemic period. The median age of the patients was 2 years, while 62.7% were under 2 years of age. Significant differences were found between the pre-COVID-19 and COVID-19 periods in terms of the number of emergency department visits (*p* = 0.003), the duration of emergency observations (*p* = 0.001) and the rates of patient visits from urban or rural centers (*p* = 0.001). Laboratory parameters, including blood sodium levels (*p* = 0.02), neutrophil counts (*p* = 0.02), base excess (*p* = 0.03), and bicarbonate levels (*p* = 0.05), also showed significant differences. Factors influencing hospitalization were found to be emergency department observation time (hours) with an OR of 0.91, 95% CI (0.867–0.974) (*p* = 0.005), blood glucose levels with an OR of 1.03, 95% CI (1.02–1.05) (*p* = 0.002), serum potassium levels with an OR of 2.36, 95% CI (1.14–4.87) (*p* = 0.02), and CRP levels with an OR of 1.02, 95% CI (1.01–1.03) (*p* = 0.006).

**Conclusion:**

The results of this study emphasize the need for targeted interventions to manage rotavirus infections, particularly in the context of ongoing public health challenges. Understanding the COVID-19 pandemic’s effects on rotavirus epidemiology is crucial for developing effective strategies to mitigate severe outcomes in vulnerable pediatric populations.

## Introduction

Rotavirus infections constitute a significant public health concern, particularly among children under five years of age, in whom they are a leading cause of severe gastroenteritis (1). These infections, characterized by high morbidity and mortality rates, impose a substantial socioeconomic burden due to their fecal-oral modes of transmission, which are strongly influenced by environmental and sanitation conditions (2). The virus typically spreads via the fecal-oral route, with an incubation period of 24–48 h (3). Nearly all children experience at least one episode of viral gastroenteritis by the age of five, and rotavirus accounts for a significant proportion of hospitalizations and outpatient visits. It is estimated that rotavirus leads to over 3.2 million outpatient visits annually in children under 5 years old (4, 5).

The COVID-19 pandemic prompted the widespread adoption of nonpharmaceutical measures, including social distancing, hand hygiene, and mask usage. While these interventions were primarily designed to mitigate the spread of SARS-CoV-2, they also inadvertently altered the transmission patterns of other infectious agents, such as rotavirus (6–8). Consequently, these preventive measures have significantly impacted various health aspects in children, particularly the incidence and management of rotavirus infections (9).

Understanding the impact of these changes is crucial. Analyzing differences in emergency department visits and hospitalizations due to rotavirus infections between the pre-pandemic and pandemic periods can provide valuable insights into how the COVID-19 pandemic influenced the disease burden created by this virus. This study aims to evaluate whether the frequency of emergency department visits for rotavirus infections varied between the pre-pandemic and pandemic periods. Specifically, it seeks to compare the rates of emergency visits for rotavirus infections before and during the COVID-19 pandemic.

## Materials and methods

This study was conducted at the Pediatrics Clinic of Gülhane Research and Training Hospital by retrospectively reviewing hospital records from the pre-COVID-19 period (January 1, 2017 – January 1, 2020) and the COVID-19 pandemic period (March 11, 2020 – August 31, 2022). It is important to note that the declaration of the onset of the pandemic in March 2020 and the lifting of mask mandates in March 2022 may have affected the circulation patterns of rotavirus during this period in Türkiye (10). Patient data were retrieved from the hospital’s electronic records system and included demographic information, presenting complaints, place of origin, number of emergency department visits, hospitalization status in the pediatric emergency clinic, treatment details, and laboratory test results.

The data of a total of 3,915 pediatric patients diagnosed with acute gastroenteritis were included in this study. The inclusion criteria required patients to be under 18 years of age and diagnosed with rotavirus infection. Patients older than 18 years old, those with a prior history of rotavirus infection, and those diagnosed with co-infections involving other enteric pathogens were excluded. The patients were categorized into two groups: those whose data were recorded during the pre-COVID-19 period and those whose data were recorded during the COVID-19 pandemic period. Factors influencing hospitalization during these two periods were analyzed to identify differences in clinical outcomes.

Stool samples were collected from patients for the diagnosis of rotavirus infection and sent to the hospital’s microbiology laboratory. All samples were appropriately packaged and then thawed for processing. Fecal specimens (1–2 mL or 1–2 g) were collected in clean, dry specimen containers to ensure that the collected virus particles were sufficient. The samples were analyzed using a rapid chromatographic immunoassay for the detection of rotavirus presence (Rotavirus and Adenovirus Combo Rapid Test Cassette [Feces], Biotest GmbH, Germany), following the manufacturer’s recommendations. The manufacturer reported the sensitivity and specificity of this test in rotavirus detection as 99.9 and > 98.8%, respectively.

The Rotavirus and Adenovirus Combo Rapid Test Cassette (Feces) is a qualitative lateral flow immunoassay designed to detect rotavirus and adenovirus presence in human fecal specimens. In this test, the membrane is pre-coated with anti-rotavirus antibodies in the R test line region. During testing, the specimen reacts with particles coated with anti-rotavirus antibodies. The mixture migrates upward on the membrane chromatographically by capillary action, reacting with the anti-rotavirus antibodies on the membrane to generate a red line. The presence of red lines in the test line region indicates a positive result, while their absence indicates a negative result.

## Statistical analysis

All data were analyzed with IBM SPSS Statistics for Windows 20.0 (IBM Corp., Armonk, NY, USA).

Numerical data determined to be normally distributed based on the results of Kolmogorov–Smirnov tests are given as mean ± standard deviation while non-normally distributed variables are given as median (minimum–maximum). For comparisons between groups, the Student t-test or Mann–Whitney U test were used in line with the normality of the considered distribution. Categorical variables are given as numbers and percentages, and inter-group comparisons were conducted with chi-square and Fisher exact tests. For the multivariate analysis, the possible factors identified with univariate analyses were further entered into the logistic regression analysis to determine independent predıctors of patient outcome. Hosmer -Lemeshow goodness of fit statistics were used to assess model fit. A 5% type-I error level was used to infer ststistical significance.

Ethical approval for the study was obtained from the Gülhane Research and Training Hospital Scientific Research Ethics Committee (2022–16/120).

## Results

A total of 3,915 pediatric patients presented with diarrhea, and rotavirus positivity was detected in 468 (6.8%) of these cases. Three hundred and forty of these rotavirus-positive cases (72.6%) were recorded during the COVID-19 pandemic period. The median age of the patients was 2 years, ranging from 1 month to 17 years. It was found that 227 (48.5%) of the patients were female, and 241 (51.5%) were male. The majority of the patients, 293 (62.7%), were aged 2 years or younger. The most common presenting complaints were diarrhea and vomiting, reported in 394 (84.2%) of the cases, while 74 (15.8%) experienced diarrhea, vomiting, and fever. A total of 408 (87.2%) patients were from the city center, while 60 (12.8%) came from rural areas (Table 1).

**Table 1:** General characteristics of patients.

Variables	*N* (%)
Pre-COVID-19	128 (27.4)
COVID-19 Period	340 (72.6)
Sex	
Female	227 (48.5)
Male	241 (51.5)
Age	
0–2 years	293 (62.7)
>2–6 years	139 (29.8)
7–18 years	36 (7.6)
Presenting complaints	
Diarrhea + Vomiting + Fever	74 (15.8)
Diarrhea + Vomiting	394 (84.2)
Place of visit	
Rural center	60 (12.8)
Urban center	408 (87.2)
Number of emergency department visits	
0–2	436 (93.1)
3 or more	32 (6.9)
Hospitalized	
Yes	83 (17.7)
No	385 (82.3)
Treatment given in emergency department	
Yes	317 (67.7)
No	151 (32.3)
	Median (min-max)
Emergency department observation time (hours)	2 (1–7)
	Mean ± SD
Blood Glucose (*n* = 261) mg/dL	79.9 ± 20.8
Blood Urea (*n* = 261) mg/dL	28.6 ± 15.7
Creatinine (*n* = 261) mg/dL	0.59 ± 0.92
AST (*n* = 261) (U/L)	50.3 ± 21.1
ALT (*n* = 261) (U/L)	29.3 ± 17.3
CRP (*n* = 261) (mg/L)	17.9 ± 45.1
Blood Sodium (*n* = 261) (mmol/L)	136.2 ± 4.33
Blood Potassium (*n* = 261) (mmol/L)	4.1 ± 0.52
White Blood Cell Count (*n* = 261) (x10^9/L)	8.65 ± 3.82
Hemoglobin (Hb) (*n* = 261) (g/dL)	12.1 ± 1.30
Platelet Count (*n* = 261) (x10^9/L)	322 ± 101
Neutrophil Count (*n* = 261) (x10^9/L)	4,408 ± 2,768
Lymphocyte Count (*n* = 261) (x10^9/L)	2,800 ± 2,268
Blood pH (*n* = 128)	7.34 ± 0.7
pO2 (*n* = 128) (mmHg)	52 ± 21
pCO2 (*n* = 128) (mmHg)	30 ± 5.6
Base Excess (*n* = 128) (mmol/L)	7.8 ± 4.4
HCO3 (*n* = 128) (mmol/L)	16.3 ± 3.8

Emergency departments were visited by 436 (93.1%) patients two times at most, while 32 (6.9%) had more than three visits. Hospitalization was required for 83 (17.7%) patients, whereas 385 (82.3%) were not hospitalized. In the emergency departments, treatment was administered for 317 (67.7%) patients, and 151 (32.3%) patients did not receive treatment. The median emergency department treatment duration of the patients was 2 h (Table 1). The laboratory test resutls of the patients are presented in Table 1.

The median age of the patients increased from 1.5 years (6 months - 17 years) in the pre-pandemic period to 2 years (1 month - 16 years) during the pandemic period (*p* = 0.20).Between the pre-pandemic and pandemic periods, significant differences were found in the numbers of emergency department visits (*p* = 0.003) and the durations of observation in the emergency department (*p* = 0.001).

In the pre-pandemic period, 18 (14.1%) of the patients arrived from rural areas, while 42 (12.4%) arrived from urban areas. Among those visiting from rural centers, 110 (85.9%) were recorded during pandemic period, and 298 (87.6%) were recorded during the pandemic period from urban centers. Significant difference was found between the two periods in terms of the rates of patient visits from urban or rural centers (*p* = 0.001) (Table 2).

**Table 2:** Comparison of rotavirus-infected patients before COVID-19 (2017–2020) and during COVID-19 (2020–2022).

Variables	Pre-COVID-19 (2017–2020) *N* (%) / Median (Min-Max)	COVID-19 Period (2020–2022) *N* (%) /Median (Min-Max)	*p*
Sex			
Female	62 (27.3)	165 (72.7)	0.98
Male	66 (66)	175 (72.6)	
Age (year)	1.5 years (6 months - 17 years)	2 years (1 month - 16 years)	0.20
Number of emergency visits (*n*)	1 (1–3)	1 (1–5)	0.003
Place of visit			
Rural center	18 (14.1)	110 (85.9)	0.001
Urban center	42 (12.4)	298 (87.6)	
Presenting complaints			
Diarrhea + Vomiting	113 (28.7)	281 (71.3)	0.13
Diarrhea + Vomiting + Fever	15 (20.3)	59 (79.7)	
Hospitalized			0.72
Yes	24 (28.9)	59 (71.1)	
No	104 (27)	281 (73)	
Treatment given in emergency department			0.14
Yes	80 (25.2)	237 (74.8)	
No	48 (31.8)	103 (68.2)	
Emergency department observation time (hours)	1 (1–24)	2 (1–72)	**0.001**
Laboratory parameters	Pre-COVID-19 (2017–2020) N (%) / Median (Min-Max)	COVID-19 Period (2020–2022) N (%) / Median (Min-Max)	
Blood glucose (mg/dL)	79 (21–142)	76 (37–184)	0.22
Blood urea (mg/dL)	24 (4–133)	27 (4–104)	0.09
Creatinine (mg/dL)	0.44 (0.34–4.40)	0.49 (0.19–14)	0.25
AST (U/L)	48.5 (17–165)	47 (16–218)	0.70
ALT (U/L)	26.5 (9–115)	26 (7–190)	0.62
CRP (mg/L)	5.35 (0.30–110)	6.1 (1–540)	0.38
Blood sodium (mmol/L)	137 (131–155)	136 (125–159)	**0.02**
Blood potassium (mmol/L)	4.2 (3–5.7)	4.1 (2.5–6)	0.44
White blood cell count (x10^9/L)	8.4 (2.4–36)	7.8 (1.6–19.20)	0.13
Hemoglobin (Hb) (g/dL)	12.2 (8.5–15.7)	12.2 (8.1–16.1)	0.94
Platelet count (x10^9/L)	314.5 (130–800)	314.5 (96–902)	0.14
Neutrophil count (x10^9/L)	4,660 (900–14,100)	3,400 (3100–13,300)	**0.02**
Lymphocyte count (x10^9/L)	2,550 (300–19,900)	2000 (300–10,800)	0.20
Blood gas parameters			
Blood pH	7.32 (7–7.43)	7.35 (7.15–7.55)	0.20
pO2 (mmHg)	58.4 (22.6–85)	47.3 (23.2–153)	0.14
pCO2 (mmHg)	26.5 (17–42)	30 (19.5–49.1)	0.06
Base excess (mmol/L)	- 6.6 (0.20–18.30)	- 8.9 (0.20–25.4)	**0.03**
Bicarbonate (mmol/L)	16.9 (8.30–28)	14.9 (6.30–23.9)	**0.05**

The bold values represent statistically significant differences between the pre-COVID-19 period (2017-2020) and the COVID-19 period (2020-2022). A p-value of less than 0.05 indicates a significant difference in the respective variable.

Presenting complaints included diarrhea and vomiting in 113 (88.3%) of the pre-pandemic patients and 281 (82.6%) of those recorded during the pandemic period, with no statistically significant difference (*p* = 0.13). Hospitalization was required for 24 (17.3%) patients in the pre-pandemic period, compared to 59 (17.4%) during the pandemic period, also showing no significant difference (*p* = 0.72) (Table 2).

Among the laboratory parameters, significant differences were observed in blood sodium levels (*p* = 0.02), neutrophil counts (*p* = 0.02), and in blood gas parameters, specifically base excess (*p* = 0.03) and bicarbonate levels (*p* = 0.05) (Table 2).

**Figure 1:**
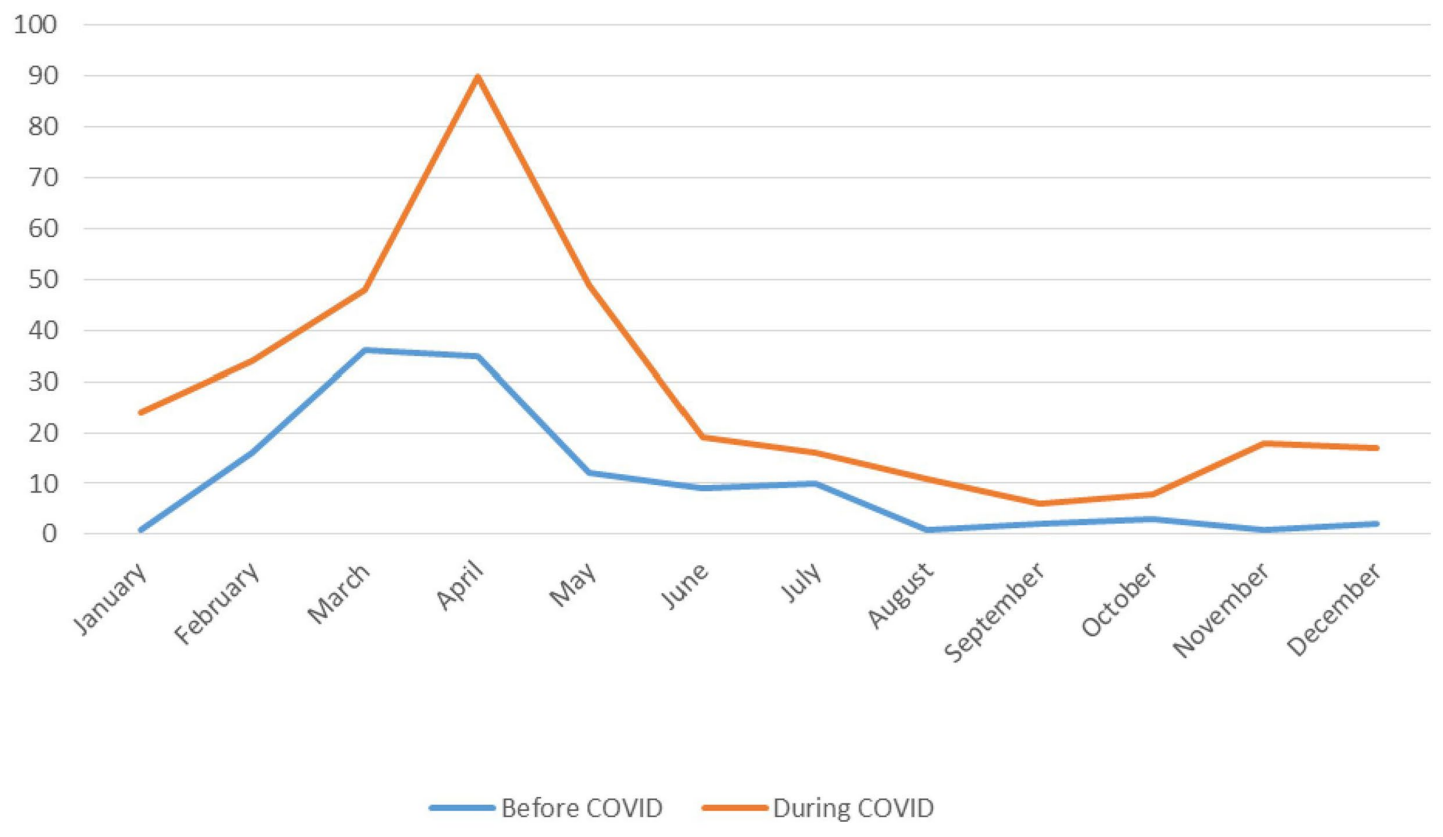
Monthly distribution of rotavirus cases before COVID-19 (2017–2020) and during COVID-19 (2020–2022).

In the comparisons made among different months of the year, it was found that rotavirus visits were more frequent from February to April during the pandemic period (Figure 1).

Model included: Age, whether treatment was provided in the emergency department, blood glucose, potassium, and CRP.

In rotavirus infections, factors influencing hospitalization were found to be emergency department observation time (hours) with an OR of 0.91, 95% CI (0.867–0.974) (*p* = 0.005), blood glucose levels with an OR of 1.03, 95% CI (1.02–1.05) (*p* = 0.002), serum potassium levels with an OR of 2.36, 95% CI (1.14–4.87) (*p* = 0.02), and CRP levels with an OR of 1.02, 95% CI (1.01–1.03) (*p* = 0.006) (Table 3).

**Table 3:** Factors influencing hospitalization in patients with rotavirus infections.

Factors	Odds Ratio (OR) 95% Confidence interval (CI)	*p*-value
Emergency department observation time (hours)	0.91 (0.867–0.974)	0.005
Blood glucose (mg/dL)	1.03 (1.02–1.05)	0.002
Blood potassium (K) (mmol/L)	2.36 (1.14–4.87)	0.02
CRP (mg/L)	1.02 (1.01–1.03)	0.006

## Discussion

This study, which included the data of 468 patients, analyzed the characteristics and outcomes of rotavirus infection. Of these, 340 (72.6%) were recorded during the COVID-19 pandemic period, while 128 (27.4%) were recorded in the pre-pandemic period. Among the patients, 62.7% were children under the age of two. The most common symptoms prompting their visits were diarrhea and vomiting combined, which accounted for 84.2% of cases. Notably, 93.1% of the patients visited the emergency department two times at most. Treatment was administered to 67.7% of the patients, and 17.7% required hospitalization. The median duration of the emergency department treatments of the patients was 2 h.

Most patients (87.2%) sought care at hospitals located in the city center. Significant differences were observed in various laboratory parameters, including blood sodium levels, neutrophil counts, base deficits, and bicarbonate levels. An increased rate of rotavirus-related admissions was noted during March and April during the COVID-19 pandemic period. No statistically significant difference in hospitalization rates was found between the pre-pandemic and pandemic periods. However, the duration of emergency department observations, blood glucose levels, serum potassium levels, and C-reactive protein (CRP) levels were identified as key factors influencing the hospitalization statuses of the patients.

Rotavirus infection is a leading cause of acute gastroenteritis in children (6). Studies have demonstrated that viruses are the primary triggers of acute gastroenteritis in children in both developed and developing countries, and rotavirus is the predominant causative agent (7, 8). Nearly all children are affected by this infection by the age of five. Research on the prevalence of rotavirus gastroenteritis among hospitalized and non-hospitalized children under 5 years of age highlighted its role as a major cause of gastroenteritis leading to severe dehydration (11, 12).

In this study, emergency department visits due to rotavirus infection were observed to be more frequent among children under the age of two, both before and during the COVID-19 pandemic. Specifically, rotavirus gastroenteritis was identified in 293 children (62.7%) aged 2 years and below, underscoring the vulnerability of this age group. Additionally, children under the age of six accounted for 90% of the total cases, emphasizing the substantial burden this infection places on younger populations.

The most common symptoms of rotavirus infection include diarrhea, vomiting, fever, and abdominal pain. Diarrhea, often watery in nature, is the predominant symptom and can result in significant dehydration, which poses a critical risk for young children (13, 14). In this study, simultaneous diarrhea and vomiting were the most frequent reasons for emergency department visits, accounting for 84.2% of the cases. A significant proportion of these visits originated from urban areas, with 87.2% of patients residing in the city center. Similarly, previous studies have reported that diarrhea occurred in 89.7% of rotavirus-infected children, while vomiting was observed in 82.5% of cases. A study conducted in Sokoto City, Nigeria revealed a higher prevalence of rotavirus infections in urban hospitals compared to rural health centers (13). This trend may be attributed to several factors, including better access to healthcare facilities in urban areas, increased awareness of symptoms that prompt earlier medical consultations, and higher population density, which can facilitate the spread of the virus. Such findings highlight the importance of understanding geographic and demographic factors in managing rotavirus infections effectively. The severity of symptoms can vary in these cases; however, rotavirus infections are particularly notable for causing acute and severe manifestations that often lead to hospitalizations (15, 16). In this study, it was found that 17.7% of the patients required hospitalization, while 67.7% received treatment in the emergency department. In particular, during the pandemic period, the duration of emergency department observations was found to be longer compared to the pre-pandemic period. Similarly, a study conducted in Poland showed an even further increase in hospitalization durations (17). These results demonstrate the significant healthcare burden posed by rotavirus, underscoring the need for targeted interventions and improved management strategies to mitigate severe outcomes. Moreover, understanding the factors that influence hospitalization durations can inform future public health policies and clinical practices aimed at managing rotavirus infections more effectively.

During the COVID-19 pandemic period, the epidemiology of rotavirus infections in children underwent substantial changes. Several studies have documented a significant decline in rotavirus activity, largely attributed to public health measures such as social distancing, mask-wearing, and schools closing aimed at controlling the spread of COVID-19. For instance, in a study conducted in Hangzhou, China, a marked decrease was reported in rotavirus and adenovirus infections among children during the COVID-19 outbreak, suggesting that these stringent measures effectively reduced the transmission of these pathogens (18). Similarly, previous research indicated an abrupt decrease in rotavirus activity during the early phases of the COVID-19 pandemic, along with a shortening of the typical seasonal patterns of rotavirus infections (19). However, in this study, the number of emergency department visits due to rotavirus infections was found to be higher during the COVID-19 period compared to the pre-pandemic period, and the duration of observations in the emergency department was also longer than those in the pre-pandemic period. These results underline a complex relationship between public health measures and rotavirus transmission, suggesting that while overall transmission rates decreased, changes in healthcare-seeking behavior or delayed access to care may have influenced the observed trends.

Rotavirus infections exhibit distinct seasonal patterns, influenced by various environmental factors and public health measures. Typically, rotavirus infections are the most prevalent during the winter months, and peaks are often observed between December and March in many regions (18). This seasonal trend is largely attributed to the resilience of the virus at colder temperatures and its enhanced transmission dynamics in crowded settings during winter months, when respiratory infections are also common (20). In this study, on the other hand, it was observed that during the COVID-19 pandemic period, the peak of rotavirus infections shifted to the spring months. Furthermore, the rate of rotavirus infections significantly increased during the COVID-19 pandemic period compared to the pre-pandemic period. There are very few studies on this topic. For instance, a study conducted in Bangladesh showed that the rate of rotavirus infections in the pediatric population rose from 23% in the period before the COVID-19 pandemic to 34% during the pandemic period (21). Similarly, Nas et al. (22) found an increase in rotavirus infection frequency during the COVID-19 pandemic period compared to the pre-pandemic period. Conversely, Li et al. (18) reported a significant decrease in outpatient visits and intestinal infections, including rotavirus and adenovirus infections, during the COVID-19 pandemic period. Additionally, a significant decrease was noted in rotavirus-related hospitalizations during both years 1 and 2 of the COVID-19 pandemic period compared to the pre-pandemic period (23). Another study highlighted an 87% drop in rotavirus cases during the implementation of COVID-19 pandemic-related restrictions, emphasizing the impact of non-pharmaceutical interventions on transmission (24). These findings not only indicate the varying impacts of the COVID-19 pandemic on rotavirus infections but also stress the importance of adapting public health strategies to effectively manage gastroenteritis in pediatric populations during such unprecedented times. This shift suggests that public health measures implemented during the COVID-19 pandemic, such as social distancing and changes in social behaviors, may have altered the typical seasonal dynamics of rotavirus transmission.

According to studies conducted so far, children with rotavirus infections tend to have longer hospital stays compared to those with other viral pathogens of gastroenteritis. Factors such as the severity of dehydration, electrolyte imbalances, and the presence of co-infections contribute to extended hospitalizations. For instance, a study conducted in Mwanza, Tanzania showed that children with rotavirus infections had a higher incidence of severe dehydration, which was directly correlated with longer hospital admissions (25, 26). Key factors influencing the severity of rotavirus infections and the need for hospitalization include sodium levels, neutrophil counts, base excess, and bicarbonate levels, as these parameters can significantly impact clinical outcomes. It was determined in this study that during the COVID-19 pandemic period, the serum electrolyte levels and neutrophil counts of the patients differed significantly compared to the pre-pandemic period. Specifically, electrolyte imbalances and neutropenia were observed more frequently among the patients during the pandemic period. The most critical factors affecting hospital admissions were the duration of observations in the emergency department, blood glucose levels, CRP levels, and potassium levels. Moreover, the pandemic may have exacerbated dehydration in children due to social isolation and the desire to avoid hospital visits to reduce the risk of infection, leading families to attempt home treatment. This avoidance of medical care could have contributed to worsening symptoms and increased the need for more intensive hospital care when these children were eventually brought to the emergency department.

The most significant limitation of this study was the lack of data on the vaccination statuses of the patients, which could have influenced the severity and outcomes of rotavirus infections. This study did not investigate the molecular characteristics of rotaviruses, specifically the P-type and G-type classifications. Understanding these classifications is crucial as they may influence both the virulence of the strains and the effectiveness of vaccines. The absence of molecular analyses limited our understanding of the genetic diversity and epidemiology of rotavirus infections in the studied population. Future research should consider incorporating molecular analyses to provide a more comprehensive understanding of rotavirus strains. Nevertheless, the strongest aspect of this study was its comprehensive analysis of clinical parameters and changes in rotavirus epidemiology during the COVID-19 pandemic period, providing valuable insights into how public health measures and healthcare-seeking behaviors have impacted the presentation and management of rotavirus infections in children.

## Conclusion

The observed unusual increase in rotavirus infections during the COVID-19 pandemic serves as an indicator not only of shifts in infection dynamics but also of changes in healthcare-seeking behaviors and the adaptations in clinical management approaches by healthcare professionals. This finding underscores the profound ways in which pandemic conditions dramatically altered both individual and healthcare system responses. Notably, delayed hospital visits may have worsened the clinical course of rotavirus infections, while healthcare professionals’ cautious approach to ruling out COVID-19 may have contributed to an increase in laboratory testing and extended observation periods. This scenario offers significant lessons on how infection dynamics and healthcare policies can be better aligned in future pandemics.

## Data Availability

The raw data supporting the conclusions of this article will be made available by the authors, without undue reservation.
